# Minimally Invasive Anterior Cervical Discectomy Without Fusion to Treat Cervical Disc Herniations in Patients with Previous Cervical Fusions

**DOI:** 10.7759/cureus.1131

**Published:** 2017-04-03

**Authors:** Robert E Jacobson, Michelle Granville, Aldo Berti

**Affiliations:** 1 Miami Neurosurgical Center, University of Miami Hospital; 2 Miami Neurosurgery Institute, University of Miami Hospital

**Keywords:** anterior cervical discectomy and fusion, adjacent-segment disease, minimally invasive anterior disc surgery, cervical nuclectomy, cervical discectomy without fusion

## Abstract

Adjacent level cervical disc disease and secondarily progressive disc space degeneration that develops years after previously successful anterior cervical fusion at one or more levels is a common, but potentially complex problem to manage. The patient is faced with the option of further open surgery which involves adding another level of disc removal with fusion, posterior decompression, and stabilization, or possibly replacing the degenerated disc with an artificial disc construct. These three cases demonstrate that some patients, especially after minor trauma, may have small herniated discs as the cause for their new symptoms rather than progressive segmental degeneration. Each patient became symptomatic after minor trauma three to six years after the original fusion and had no or minimal radiologic changes of narrowing of the disc or spur formation commonly seen in adjacent level disease, but rather had magnetic resonance imaging (MRI) findings typical of small herniated discs. After failing multiple months of conservative treatment they were offered surgery as an option. Subsequently, all three were successfully treated with minimal anterior discectomy without fusion. There are no reports in the literature of using minimal anterior cervical discectomy without fusion in previous fused patients. This report reviews the background of adjacent level cervical disease, the various biomechanical explanations for developing a new disc herniation rather than progressive segmental degeneration, and how anterior cervical discectomy without fusion can be an option in these patients.

## Introduction

This report discusses the rationale and technique for simple cervical discectomy without fusion in selected patients that developed small to moderate cervical herniated discs at a new cervical level after previous cervical fusion and instrumentation. All three patients with previous cervical fusions became symptomatic only after minor new trauma, rather than as a result of progressive changes secondary to adjacent segmental degeneration after previous cervical fusions [1-2]. All previous reports of cervical discectomy without fusion only discussed this procedure for cervical discs in patients without previous cervical fusion surgery [3-4]. With the development of more microsurgical and minimally invasive procedures that can leave part of the disc and all of the supporting structures intact, these patients now have the option of not having to undergo an extensive surgical procedure, especially when symptoms are caused by a small herniated cervical disc. In these three cases, the precipitating minor trauma occurred between three to six years after the original surgery. The patients developed persistent symptoms consisting of neck pain and minor radiation to arms with numbness to the upper extremities. Radiologic studies, including magnetic resonance imaging (MRI) scans, found a small herniated disc above the previous surgery in all three patients at the C3-4 level. Each of them had six to 14 months of various types of conservative therapy consisting of anti-inflammatory medications and physical therapy. After failing conservative treatment, surgery was discussed and there were either medical contraindications to open surgery including fusion, or the patient did not want extensive open surgery. The patients were explained the options of repeat anterior fusion or posterior decompression and they gave informed consent understanding that this was a minimal approach to the herniated disc.

It is apparent that there is a different clinical course, in addition to distinct radiologic findings, in cases which develop small to medium disc herniations above the fusion after minor trauma when compared to patients suffering from adjacent segment degeneration [2-3]. The accepted mechanical theories for adjacent segment degeneration include increased motion above the fusion. Hypermobility is more commonly seen after multi-segment fusions as the cause of adjacent level cervical degeneration. There are experimental and clinical observations that demonstrate that development of a new herniated disc, possibly secondary to increased intradiscal pressure above a previous fusion, may be another mechanism for new symptoms in patients that have undergone previous cervical fusion, rather than mechanical hypermobility [5-6]. There are very few studies emphasizing the possible role a new herniated disc plays after previous cervical fusion [5]. These cases had minor trauma that was superimposed on possibly altered intradiscal changes due to the previous cervical fusion which makes the disc more vulnerable to herniation. It is unclear if this represents separate disc pathology or is a precursor of later post fusion segmental degeneration.

## Technical report

### Patient characteristics

**Figure 1:**
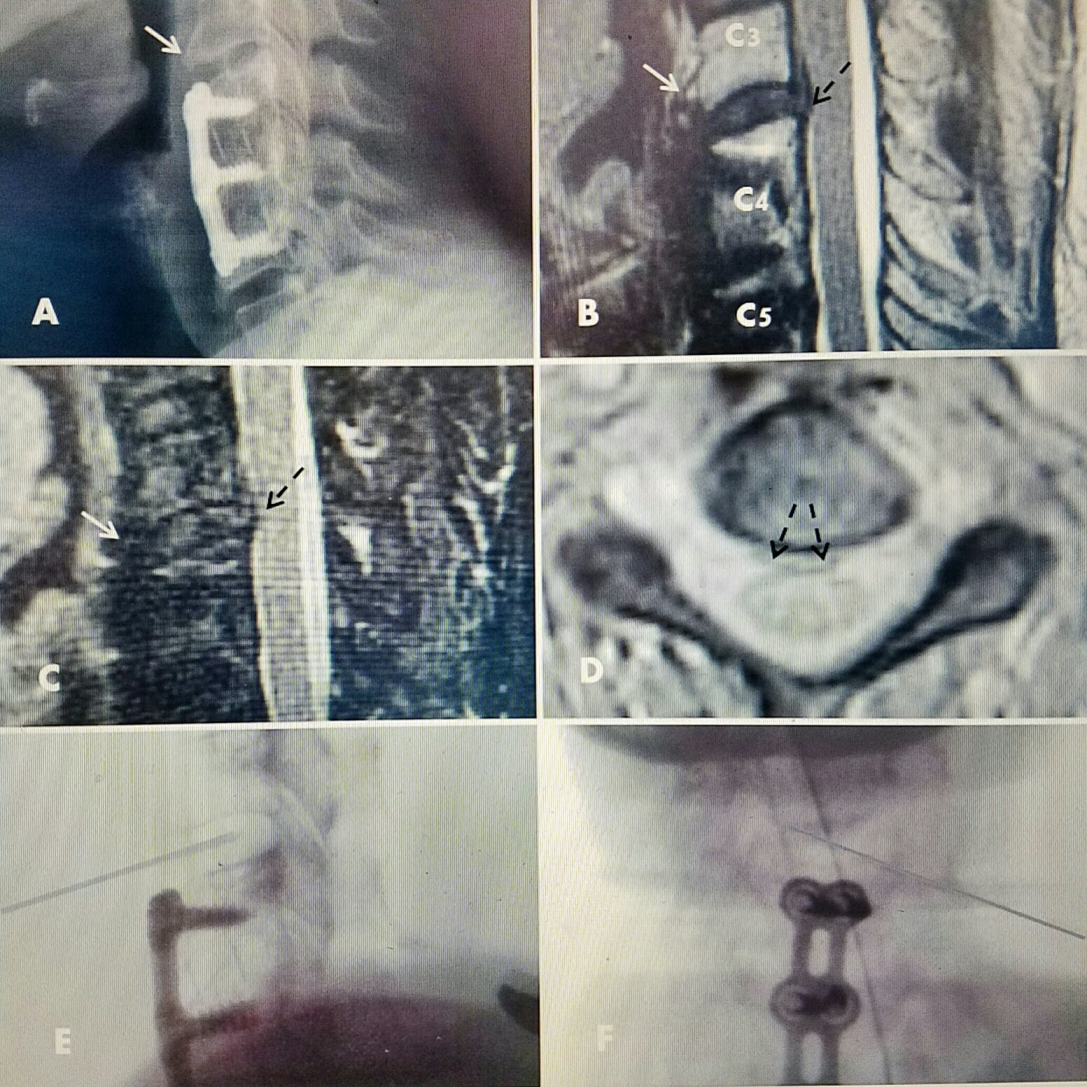
Patient #1 with C3-4 herniated disc above previous C 4-C 6 fusion A: Plain X-rays showing solid C4-C6 ACDF with screws and plate with interbody fusion performed five years previously. Normal C3-4 alignment and disc height. Beginning anterior spur C3 (white arrow). B: T2 MRI showing C3-4 herniated disc (dotted black arrow) without endplate change or narrowing but beginning ventral spur anteriorly C3 (white arrow). C: MRI Sagittal STIR image: clear C3-4 disc herniation (dotted black arrow) with small superior extension behind C3 vertebral body. D: Axial T2 MRI, small C3-4 disc herniation midline and slightly to right of midline (dotted black arrows). There are no bone or degenerative changes in the foramina or facet joints and no foraminal narrowing. E/F: 1.3 mm discectomy probe in C3-4 disc space. ACDF: Anterior cervical discectomy and fusion; MRI: Magnetic resonance imaging.

**Figure 2:**
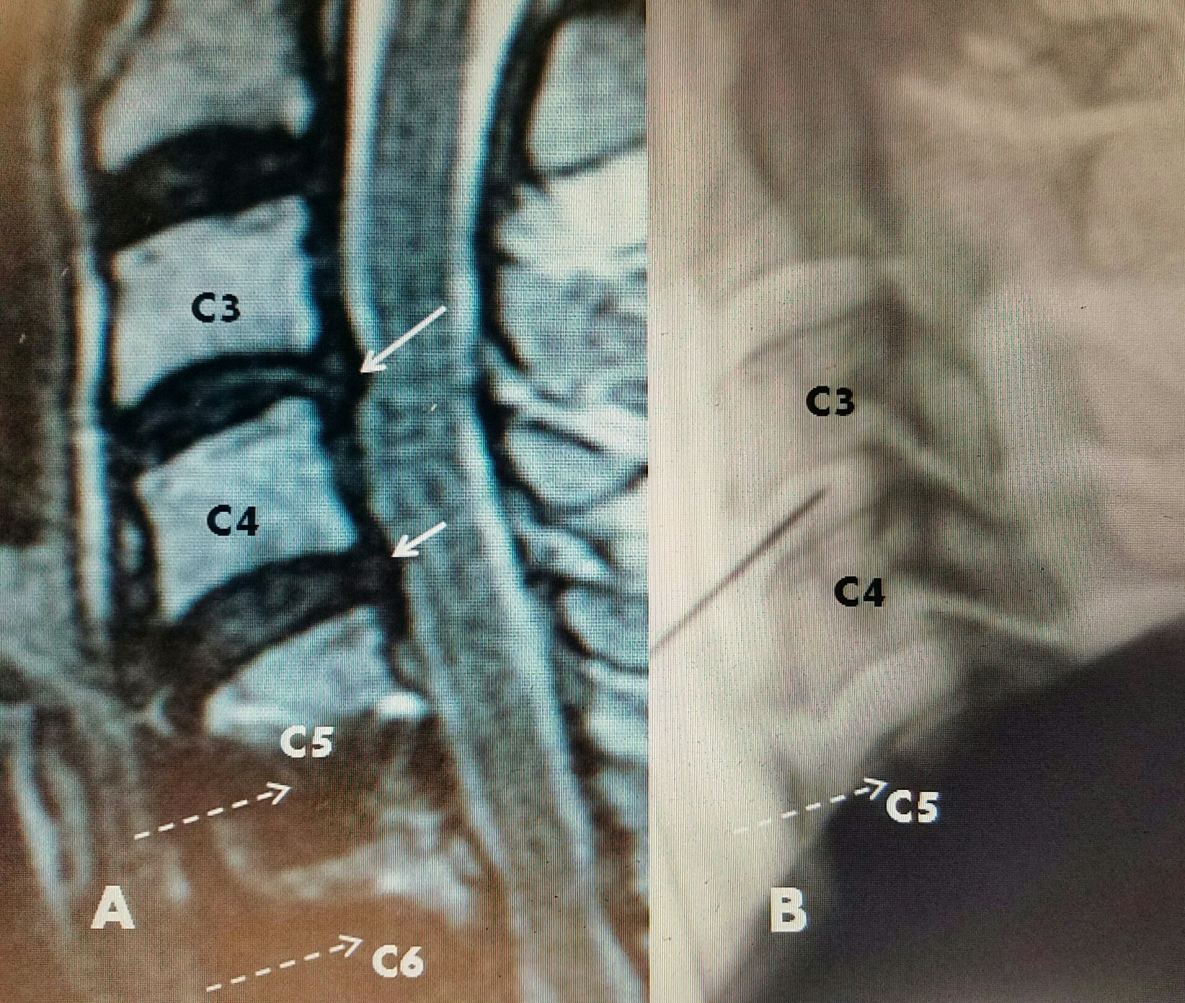
Patient #2: Previous C5-C7 ACDF and plating, new C3-4 disc A: T2 sagittal MRI: 2-3 mm C3-4 disc herniation (long white arrow) and smaller C4-5 protrusion (short white arrow). C5 and C6 anterior screws below (dotted white arrows). B: Intraoperative picture of discectomy probe within C3-4 disc space. The probe can move within the disc space making contact with both superior and inferior endplates. Debridement can be performed with endplate contact. ACDF: Anterior cervical discectomy and fusion; MRI: Magnetic resonance imaging.

**Figure 3:**
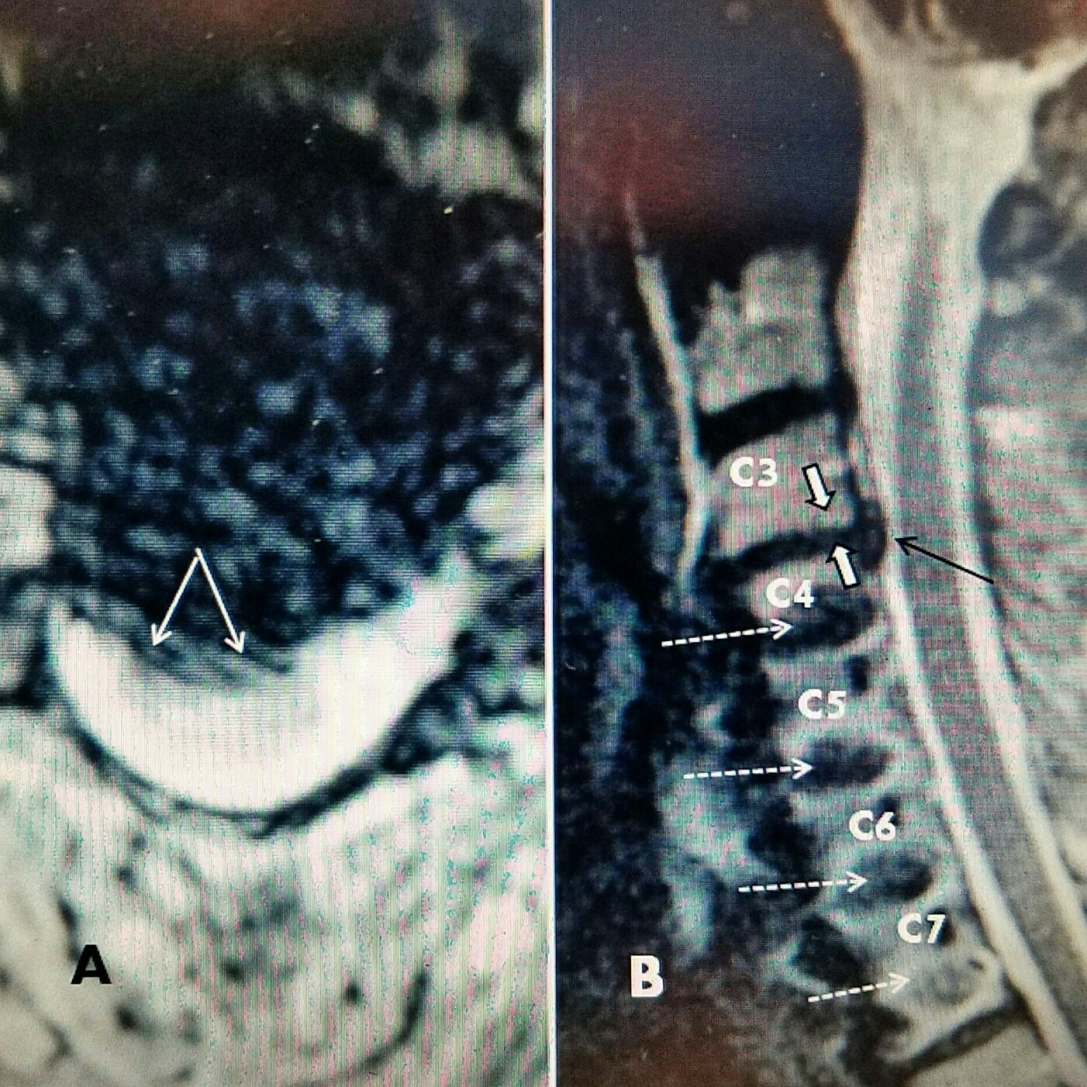
Patient #3 C3-4 herniated disc above previous ACDF and plating C4-C7 A: Axial MRI: C 3-4  midline and right lateral herniated disc (two solid white arrows). B: Sagittal T2 MRI shows 3 mm herniated C 3-4 disc (black arrow) with some moderate posterior disc space narrowing (white arrows). Screws at C4, 5, 6, 7 (dotted arrows). ACDF: Anterior cervical discectomy and fusion; MRI: Magnetic resonance imaging.

All the three patients had a course of six to 14 months of conservative treatment prior to any consideration for surgery (Table 1). The first patient, a 57-year-old male, had his original anterior cervical discectomy and fusion (ACDF) with interbody graft and screws with plating from C4-C6, five years prior to his new injury. Subsequently, he had coronary artery surgery three years after his C4-C6 cervical fusion. His new symptoms developed after a 'whiplash' accident. He persisted with pain despite six months of conservative treatment and an MRI showed a small C3-4 disc immediately above the original surgery (Figure 1). After preoperative medical evaluation, he was felt to be a high risk for extensive cervical surgery due to his cardiac issues, so he was offered the option of minimal discectomy which was performed with a cutting extracting instrument (Stryker DeKompressor®, Denver, CO, USA ) [7]. The second patient (Figure 2) had a C3-4 disc above a previous C5-C7 fusion six years earlier and had surgery performed with the simple cutting extracting decompressor, while the third patient (Figure 3) had a C3-4 disc three years after a C4-C7 fusion. This last patient had a manual discectomy through a larger 2.9 mm tubular cannula allowing for disc removal with the pituitary rongeur. The larger tube was used because of the size of the disc on MRI scan, minimal posterior disc space narrowing, and the plan to partially curette the endplates after discectomy. All patients were explained the difference of the minimal procedures from open discectomy with fusion or cervical decompression and they gave informed consent.

**Table 1:** Patient information

Patient	Age	Sex	Original cervical surgery	New injury	Interval from original surgery	Symptoms	Conservative RX time	New disc	Technique
#1	57	M	C4-C6 Ant fusion + plating	Auto	5 years	Neck + bilateral arm pain	6 months	C3-4	Cut/aspirate
#2	49	F	C5-C7 Ant fusion + plating	Fall	6 years	Neck and hand numbness	14 months	C3-4	Cut/aspirate
#3	55	F	C4-C6 Ant Fusion + plating	Auto	3 years	Neck and right arm pain	8 months	C3-4	Tubular manual
									Discectomy

### Surgical technique

All patients had surgery performed in an ambulatory surgery center and were discharged home the same day after surgery. Patients were given 1 gm of cephalin or 600 mg of clindamycin intravenous before surgery. Under general intubation anesthesia, the endotracheal tube was secured to the right side creating slight lateral traction on the trachea to the right. A roll was placed under the shoulders with the neck in a slightly lordotic position. The shoulders were then lightly taped down since the C3-4 interspace was easy to visualize under lateral fluoroscopy. With a lateral view, the C3-4 disc was identified, the skin marked, and anteroposterior (AP) fluoroscopy was used to again count and visualize the disc space. The c-arm fluoroscopy was angled to get a clear view of the uncovertebral joint and assure the spine was aligned perfectly straight. The previous plate and screws were identified. In two cases the C3-4 disc was the superior adjacent disc space and in the third case, the abnormal space was also at C3-4, but two spaces above the previous cervical plate.

**Figure 4:**
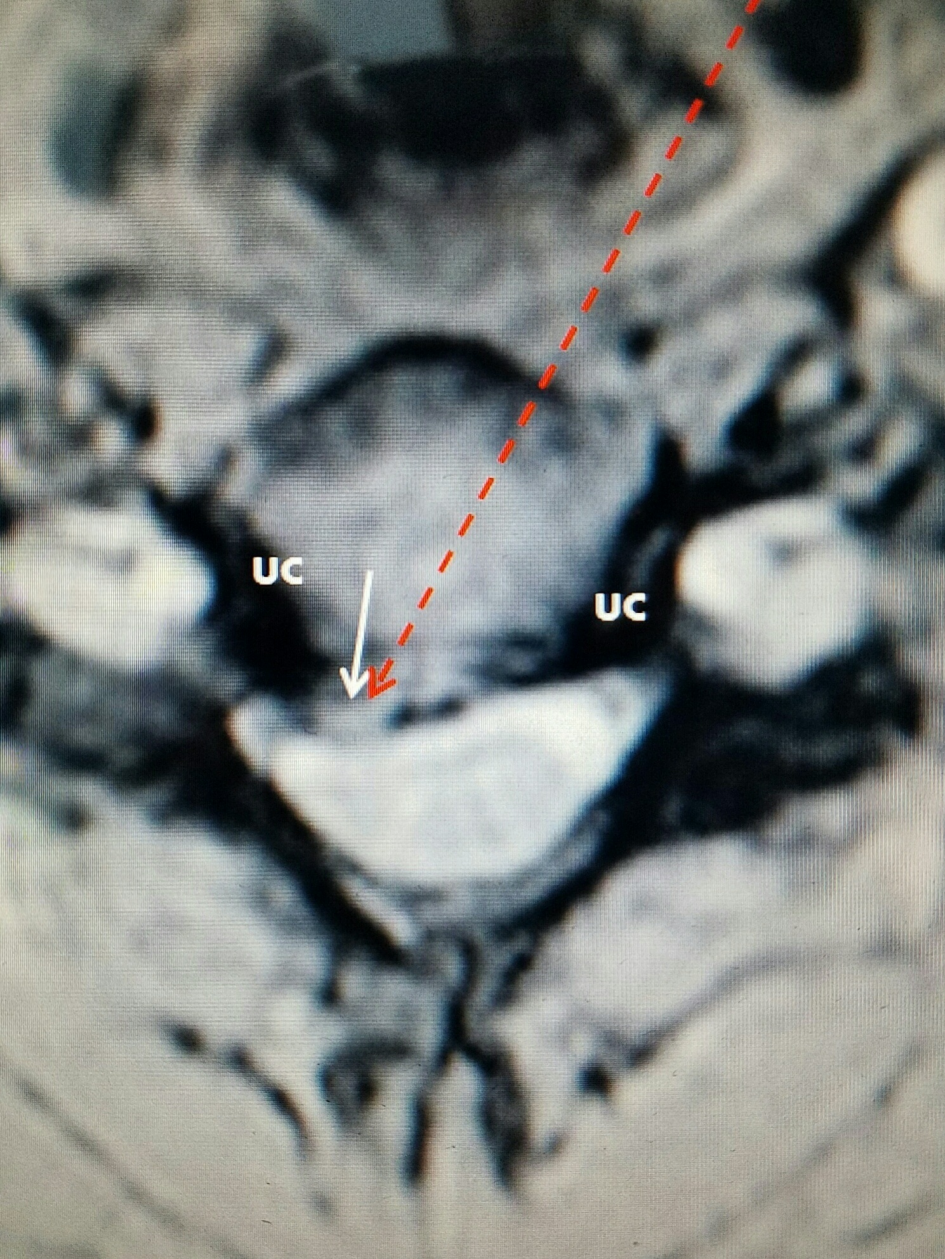
Patient #1: Axial T2 MRI of herniated disc at C3-4 to right The small white arrow shows the right posterolateral disc herniation. The darker lateral uncovertebral joints (UC) appear normal and keep the discectomy instruments confined within the central and paracentral regions of the disc space. The dashed red arrow is the percutaneous path for the access cannula to enter the disc. It is positioned to move across the disc space to the herniation. MRI: Magnetic resonance imaging.

The skin was then prepped and draped. All cases were approached from the left side of the neck. A 20 gauge spinal needle was used under lateral fluoroscopy to align parallel to the C3-4 disc space. AP fluoroscopy was then used to mark just medial to the uncovertebral joint, which was the initial entry point for all instrumentation within the disc. With direct finger pressure, the left sternocleidomastoid and carotid sheath were palpated and using light finger pressure the carotid sheath was pushed slightly laterally under the sternocleidomastoid muscle. At the same time, the trachea (with the endotracheal tube slightly across the midline) was pushed slightly medially creating direct contact with the anterior cervical vertebrae. At this point with continuous pressure the prevertebral space and the anterior cervical spine could be felt in all cases. For maintaining constant pressure the skin was then anesthetized with one percent lidocaine and for keeping continual finger pressure pushing the carotid sheath laterally and the tracheal across the midline, a fine 20 gauge spinal needle was directed under fluoroscopic guidance into the C3-4 disc space. The initial view was AP so that the uncovertebral joint on the operative side could be easily identified. After entering the anterior annulus of the disc, a lateral view was obtained to: 1) make sure it was the C3-4 disc and 2) make any adjustments in angle to ensure entry was aligned to the middle of the disc in the sagittal view and parallel to both the superior and inferior endplates of C3-4, since the surgical approach is based on the axial anatomy of the cervical disc space (Figure 4). The same anatomic approach was used on all the three patients that had herniation at the C3-4 disc.

Once proper position was confirmed, then a slightly larger – either 1.6 mm or 2.9 mm – cannula was introduced directly parallel to the spinal needle to enter through the anterior annulus and capsule of the disc. Two different methods were used.

**Figure 5:**
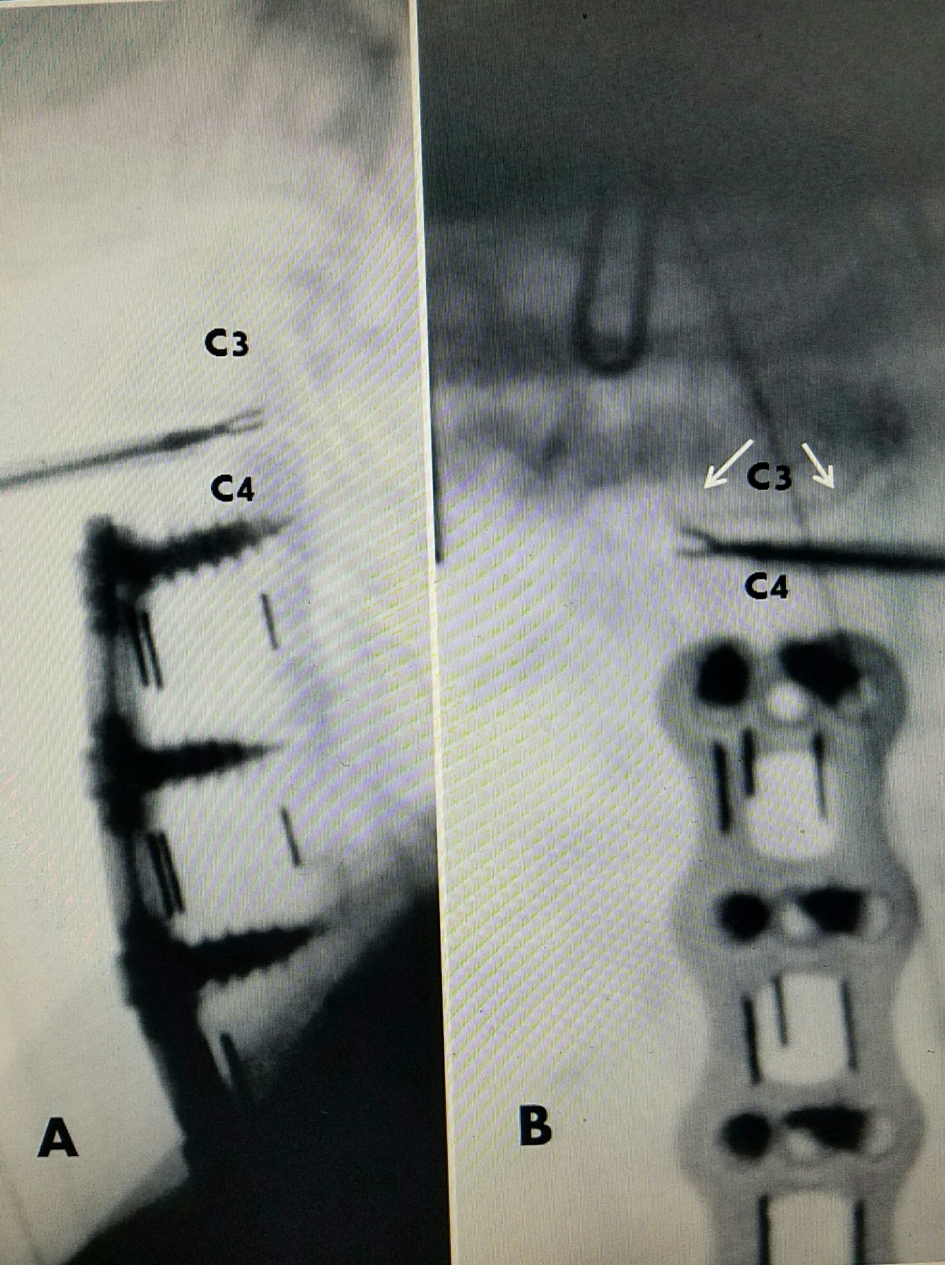
Patient #3: Microtubular manual C3-4 discectomy A: Lateral operative picture showing 2.9 mm cannula with 2.5 mm pituitary rongeurs removing posterior-lateral disc at C3-4 above previous fusion and plating C4-C7. B: AP view showing cannula entering C3-4 disc on patient's left and crossing midline so that pituitary rongeurs is removing disc from right posterior part of the disc space. Uncovertebral joints (two solid white arrows). Cannula enters just medial to UC. The UC prevents pituitary from going too far lateral and confines instrument to disc space. AP: Anteroposterior.

1) Two cases were performed with the Stryker Dekompressor® which is a cutting extracting 16 gauge probe that removes disc material based on an Archimedes screw mechanical principle [7]. It is 3 inches in length and once inserted in the disc the fine cannula can be easily moved under fluoroscopic guidance around the soft nucleus of the disc removing disc material. After this was done, the disc space was irrigated with 1 or 2 cc of normal saline which frees up further parts of the nucleus that are then removed. The probe was slowly moved both posteriorly and across to the opposite side which targets the disc herniation. In all cases, as more disc material is removed, the cannula could be moved toward the endplate. The cannula and probe can be maneuvered to the endplates and if continued the instrument can grasp and debride adjacent to the endplates. When no further disc material was removed, the procedure was terminated.

2) Because of the larger size of the disc on MRI scan and slight posterior disc space narrowing this case was performed using a 2.9 mm cannula placed by the same steps and identical anatomic position. After the 20 gauge needle was positioned while maintaining manual finger pressure on the prevertebral area, a guidewire was placed into the disc adjacent to the needle. Next, under fluoroscopic guidance, the cannula was then slid down to the anterior annulus and twisted into the annulus to a depth of 2-3 mm so that it maintains position in the annulus. Sequentially, a small manual 1.5 mm bone drill was manually used under fluoroscopy to perforate the remainder of the annulus just into the anterior 1/3 of the disc space under both lateral and AP fluoroscopy. Following this, 2 and 2.5 mm fine curette and pituitary rongeurs were used to loosen and remove disc material (Figure 5). The instruments moved obliquely across the disc space toward the posterolateral herniation. Finally, the superior and inferior endplates were lightly curetted using a combination of the surgeons' manual feel and radiographic confirmation of the position of the curette being either on the superior or inferior endplate.

**Figure 6:**
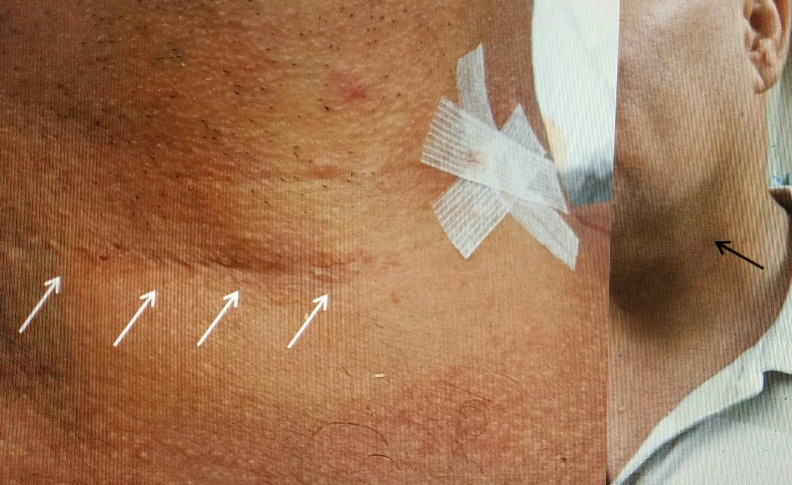
Comparing previous ACDF incision to minimally invasive incision Patient #1 with original right lower cervical incision for C 5-7 ACDF and plating (four solid white arrows). Tape for minimally invasive surgery at C 3-4 approached from left. Far right picture three weeks postoperative. Minimal scar (solid black arrow). ACDF: Anterior cervical discectomy and fusion.

Before withdrawal of the cannulas, 20 mg depomedrol is injected in the anterior annulus and 20 mg in the prevertebral space. The punctate skin wound was closed with tape without stitches (Figure 6). All patients were given a firm cervical collar to wear continuously for the first four-seven days after surgery. They were given a muscle relaxant to take at night as needed. If they had no pain in five days, they removed the collar. The initial postoperative visit was between seven and 10 days after surgery. Patients could resume normal activities and were not routinely prescribed physical therapy. All patients had minimal pain postoperatively, only wore the collar for the prescribed five days, and took minimal medication. Both patients that were working prior to surgery returned to previous employment within 7-10 days. The third patient that did not work resumed housework.

## Discussion

The development of adjacent spinal segment degeneration is a common problem seen after successful single or multilevel cervical fusions [1]. However, studies show it can also be seen after simple discectomy without fusion and also in follow-up radiologic studies in asymptomatic patients after previous cervical fusions [2-3]. For many years the prevailing concept was that successful fusion and rigid immobilization of the cervical spinal segments led to a 'hypermobility' of the segment above [2]. This hypermobility at the superior adjacent segment was attributed to be the main cause of adjacent segment degeneration (ASD) defined as the radiologic finding and distinguishing from adjacent segment disease (ASDis) when a patient was symptomatic. These plain radiologic changes include adjacent disc space narrowing, spur formation, mild kyphotic angulation, and possibly micro movement with flexion and extension. MRI scans demonstrate similar disc space narrowing with prominent T2 signal changes within the disc space suggesting fibrotic replacement of soft disc. There is also ligamentous thickening, fibrous disc protrusion and in more advanced stages, posterior facet hypertrophy and axial and sagittal canal stenosis [2-4].

Recently, many large reviews have questioned if the degenerative disc process identified by radiologic changes above a previous fusion is just the natural progression of cervical disc disease, rather than a sign of hypermobility. Multiple studies and literature reviews show there is a cumulative three percent increase in radiologic disease and a 1.9% increase in clinical disease yearly. Kong, et al. found that in the presence of radiologic ASD less than 50% of the patients were symptomatic, but that number increased 1.4% per year [2]. The literature focuses on the progressive symptomatic nature of the disease as well as the possibility that some percentage of later identified adjacent level disease was actually minor preexisting degenerative changes that were present at the time of the original cervical surgery, but were felt not to be significant at the time and not included as an additional level at the time of surgery [3-4]. In this same series of patients, a long-term retrospective review found new symptoms and findings developed in previously operated asymptomatic patients after new 'trauma', more commonly in patients five or more years after the original surgery, while adjacent level symptoms often appeared within the first two years [3]. This is similar to our findings in that all the three patients who had small C3-4 disc herniation developed symptoms between three and six years after the initial multilevel cervical fusion. Long-term studies clearly show a statistical yearly progression with or without fusion.

**Figure 7:**
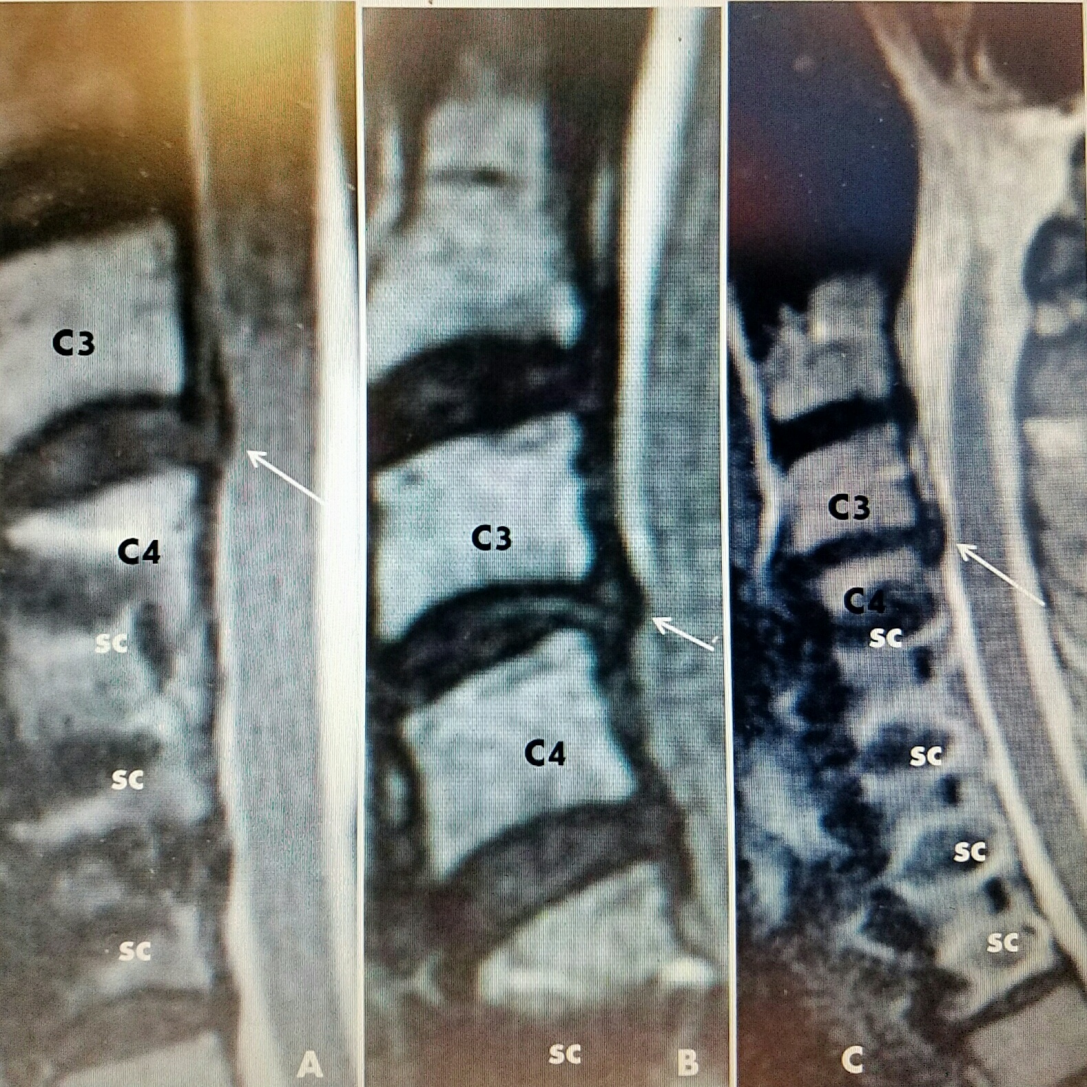
Sagittal MRI scans of three cases A: Case #1: previous C4-C6 fusion and plating with screws (sc), small C3-4 disc adjacent level (white arrow). B: Case #2: C5-C7 fusion and screws (sc) and small herniated disc C3-4 (white arrow). C: Case #3: Fusion with screws C4-C7 (sc) and slight disc narrowing and subligamentous herniation C3-4 (white arrow). MRI: Magnetic resonance imaging.

Biomechanical studies show that the C4-5, C5-6, and C6-7 segments are the most mobile, the most frequent to have a radiologic finding of herniated disc, and the most frequently fused levels [5]. The remaining upper cervical segments are actually less vulnerable to being aggravated by motion normally but after fusion at C4 or below, the upper cervical disc levels are the only remaining segments with motion. Biomechanical testing shows that the intradiscal pressure in cervical discs above fusions is elevated compared to non-fused discs during in vitro testing [5-6]. The hypermobility above the fusion combined with increased intradiscal pressure in the remaining unfused discs is a partial explanation as to why all three of our cases developed new herniated discs at the C3-4 level (Figure 7).

Along with hypermobility, biomechanical analysis has also demonstrated that sagittal alignment post fusion is a factor [4-6]. In the composite picture of all the three patients, the MRI scans all show normal sagittal alignment. It has also been suggested that the external anterior cervical fusion plate may cause impingement on the unfused disc above and could place pressure on the previously normal disc above the fusion just with normal spinal motion [7-9]. However, when frequencies of adjacent level findings in patients with plate systems are compared to cases with zero profile constructs, there is a similar statistical incidence of degeneration indicating the plate is not a significant mechanical factor affecting the superior adjacent disc [10-11]. Patients with two or more levels of fusion have an increased mobility compared to single level fusion [5,12]. Avoiding this rigid fixation with the development of artificial disc replacement has allowed some preservation of cervical spine motion. Mid and long-term studies of one and two-level artificial cervical disc replacements show reduction, but not elimination of the incidence of adjacent level disc by 50% [13]. This indicates that avoiding absolute rigidity may diminish the hypermobility that develops over time in disc segments above solid cervical fusions.

Generally patients with symptomatic ASD, if conservative measures fail to relieve their symptoms, are faced with the possibility of either extensive surgery involving adding another level of fusion, posterior laminectomy and instrumentation, revision of the previous instrumentation below the new cervical disc degeneration and extending the fusion to the new adjacent level, or placement of an artificial disc construct to preserve motion [9,11]. In patients with previous fusions, these surgical options are technically challenging and have risks and complications because of scar formation and distorted anatomy in the prevertebral fascia and anterior cervical space. There are no reports in the literature discussing simple cervical discectomy without fusion as an option for treating patients with previous adjacent cervical fusion. This background information indicates that if a previous cervical fusion patient develops a small disc herniation without associated disc narrowing after many years, then simple discectomy without fusion is a reasonable approach.

Simple cervical discectomy and nuclectomy have been frequently and widely performed with either cutting extractors, lasers, or combined with using endoscopes or small cannulas and pituitary rongeurs [14]. There are numerous reports in the literature of performing simple anterior discectomy or nuclectomy without fusion for one or two level contained herniated discs. In follow-up, a significant percentage of these simple discectomies will actually go on to fuse the disc space. Recently, experience with advanced endoscopic cervical surgery allows for removal of small uncovertebral spurs and performance of interbody fusion [15]. However, fusion often occurs with some disc space narrowing, especially if the endplates have been debrided, compared to the slight widening due to the interbody spacer with an interbody fusion [16-17]. Minimal cervical discectomy with endplate debridement increases the fusion rate [14-15,18].

These three cases emphasize that a small disc herniation should be considered in addition to adjacent segment degenerative disease in a patient that becomes symptomatic after minor trauma and had previous cervical spinal fusion or corpectomy, especially if there is a prolonged multiyear asymptomatic interval [3]. Minimally invasive disc resection above a previous fused and instrumented cervical segment is possible if there is a small disc herniation rather than segmental degeneration. By only decreasing the protruding part of disc while leaving the remaining nucleus through a minimal incision in the anterior annulus may leave the affected disc and the disc segment more structurally stable.

## Conclusions

In all three cases, the patients developed new symptoms after minor cervical trauma, three to six years after the original multilevel fusions with instrumentation. The new herniated discs were at the same C 3-4 level above the previous fusions. Radiographically, both with MRI scans and flexion and extension films, these small herniated discs were not directly related to the typical radiologic signs of adjacent segment degeneration. This correlates with biomechanical studies presented show that previously normal discs may be more vulnerable to herniation after fusion at nearby segments. This possibly can be another factor in the mechanisms besides hypermobility which ultimately leads to adjacent segmental degeneration years after a cervical fusion. All the three patients were treated with minimal cervical discectomy without fusion with symptomatic relief over a 12 to 30-month follow-up. Minimally invasive cervical techniques are an option for patients that are shown to have only disc herniation, rather than segmental degeneration after minor trauma and previous cervical spinal fusions, or have significant medical contraindications to more major open cervical surgery.

If a patient with previous multilevel cervical fusion and plating develops new symptoms after minor trauma besides consideration of adjacent segment degeneration, a new cervical disc herniation above the fusion must also be considered. New symptoms may not be an absolute sign of mechanical problems from a previous fusion. This report demonstrates that it is both technically possible and clinically effective to utilize simple minimally invasive discectomy without fusion to provide symptomatic relief in specific cases.
